# Impact of severity of Covid‐19 infection on cognitive function: Characterisation of the BEACON trial cohort

**DOI:** 10.1002/alz70857_105276

**Published:** 2025-12-25

**Authors:** Abbie Palmer, Millie Sander, Clive G Ballard, Steve Lewis, Shanice Tulloch, Arit Inok, Dag Aarsland, Adam Hampshire, Anne Corbett

**Affiliations:** ^1^ University of Exeter, Exeter, United Kingdom; ^2^ Institute of Psychiatry, Psychology & Neuroscience, King's College London, London, United Kingdom; ^3^ Imperial College London, Department of Brain Sciences, London, London, United Kingdom

## Abstract

**Background:**

Long Covid affects at least 9.9% of people. Neuropsychiatric symptoms are common, with cognitive impairment affecting at least 25% of patients. Cognitive deficits impact on individuals’ employment, social activity, wellbeing and quality of life. They are also a key risk factor for onward cognitive impairment and dementia particularly in older adults. However, the aetiology of these cognitive symptoms of Long Covid remains relatively poorly understood. This poster reports on baseline data from BEACON, a trial cohort of people with Long Covid, and provides comparative analysis with data from the PROTECT‐UK cohort of older adults.

**Method:**

2185 participants with subjective cognitive impairment following Covid‐19 infection were recruited to the online BEACON trial. Participants completed demographic and health‐related questionnaires including mental health, fatigue and wellbeing. They also completed the validated FLAME cognitive test system of eight cognitive tests of memory, attention and executive function. Descriptive statistics and t‐test analysis were used to characterise the BEACON cohort cognitive profile, and regression analyses examined factors associated with cognition. An age‐matched dataset was generated from the PROTECT‐UK data from participants with no history of Covid‐19 for comparative analysis of cognitive performance.

**Result:**

Analysis of BEACON cognitive data demonstrated a unique profile of cognitive impairment compared with healthy controls, with particularly marked impairment in memory and executive function in individuals with a history of more severe Covid‐19 infection. Regression analysis showed associations of lifestyle factors with cognitive impairment.

**Conclusion:**

This analysis provides a detailed cognitive characterisation of patients with Long Covid, highlighting potentially unique cognitive trajectories in these individuals. These findings emphasise the importance of providing clear, specific clinical guidance for detection, monitoring and treatment of cognitive impairment in this patient group.

